# Electronic Northern Analysis of Genes and Modeling of Gene Networks Underlying Bovine Milk Fat Production

**DOI:** 10.1155/2017/1910530

**Published:** 2017-11-07

**Authors:** Bhaskar Ganguly, Tanuj Kumar Ambwani, Sunil Kumar Rastogi

**Affiliations:** Animal Biotechnology Center, Department of Veterinary Physiology and Biochemistry, College of Veterinary and Animal Sciences, G. B. Pant University of Agriculture and Technology, Pantnagar 263145, India

## Abstract

Milk fat is one of the most important economic traits in dairy animals. Yet, the biological machinery involved in milk fat synthesis remains poorly understood. In the present study, expression profiling of 45 genes involved in lipid biosynthesis and secretion was performed using a computational approach to identify those genes that are differentially expressed in mammary tissue. Transcript abundance was observed for genes associated with nine bioprocesses, namely, fatty acid import into cells, xenobiotic and cholesterol transport, acetate and fatty acid activation and intracellular transport, fatty acid synthesis and desaturation, triacylglycerol synthesis, sphingolipid synthesis, lipid droplet formation, ketone body utilization, and regulation of transcription in mammary, skin, and muscle tissue. Relative expression coefficient of the genes was derived based on the transcript abundance across the three tissue types to determine the genes that were preferentially expressed during lactation. 13 genes (*ACSS1*,* ACSS2*,* ADFP*,* CD36*,* FABP3*,* FASN*,* GPAM*,* INSIG1*,* LPL*,* SCD5*,* SPTLC1*,* SREBF1, *and* XDH*) showed higher expression in the mammary tissue of which 6 (*ADFP*,* FASN*,* GPAM*,* LPL*,* SREBF1,* and* XDH*) showed higher expression during adulthood. Further, interaction networks were mapped for these genes to determine the nature of interactions and to identify the major genes in the milk fat biosynthesis and secretion pathways.

## 1. Introduction

Milk fat content is regarded as one of the most important economic traits of milch animals; identification of gene networks that regulate lipid biosynthesis and secretion in the mammary gland is essential to our understanding of lactation physiology. Finding candidate genes for improved fat content represents a constant research goal [1] that may further provide opportunities for genetic manipulations to derive more or better milk fat. Comparing biomolecular composition of mammary tissue with other tissues can allow insights into the molecular responses that govern milk fat production. Transcriptional regulation is a major long-term mechanism for the control of metabolism, and switching on and off gene expression essentially drives a cell's biological function and activity [2]. In the present study, an attempt has been made to identify the genes, which are differentially expressed during milk fat production in bovines, and determine their interaction networks using a computational approach.

## 2. Materials and Methods

### 2.1. Identification of Differentially Expressed Genes

The reference bovine gene sequences for 45 genes, previously known to be involved in lipid synthesis (Table 1) [3], were obtained from Ensembl [4]. Electronic Northern (*e*-Northern) was performed using dbEST and UniGene; briefly, the dbEST [5] was queried for these sequences by BLASTN* v*2.2.27 [6] using default parameters and the significant hits were looked up in UniGene ESTProfile [7] for transcript abundance based on normalized “transcripts per million” (TPM) values in mammary tissue (TPM_ma_), skin (TPM_s_), and muscles (TPM_mu_). Where information was available, transcript abundance (value not shown) was also compared between adult and young stages.

Percent mammary transcript abundance for a gene *x* was calculated using the formula:

(1) %  Transcript  abundance=TPMmax∑TPMma  all  genes×100.

To confirm preferential mammary expression, relative expression coefficient (*E*_*r*_) was calculated as the ratio of mammary transcript abundance to the geometric mean of cutaneous and muscle transcript abundance; that is,

(2) $$\begin{array}{c} E_{r}=\frac{{TPM}_{ma}}{\surd \left({TPM}_{s}\cdot {TPM}_{mu}\right)}. \end{array}$$

A twofold change in *E*_*r*_ was, arbitrarily, assumed to be significant; that is, upregulation of expression was inferred when TPM_ma_ > TPM_s_ and *E*_*r*_ ≥ 2. Similarly, downregulation was inferred when TPM_ma_ < TPM_s_ and *E*_*r*_ ≤ 0.5.

### 2.2. Gene Network Analysis

Interaction networks and coexpression profiles for the genes were derived using STRING* v*9.1 with default settings [8]. STRING is a web-based application for network generation and visualization that uses a database of physical and functional protein interactions derived from four separate sources, namely, genomic context, high-throughput experimental data, coexpression, and existing literature. It quantitatively combines the information from these four sources to generate a weighted interaction network.

## 3. Results and Discussion

### 3.1. Transcript Abundance

Transcript abundance was inferred from UniGene ESTProfile on the basis of normalized TPM values (Table 2) for the 45 genes involved in nine bioprocesses including fatty acid import into cells* (CD36*,* LPL*, and* VLDLR)*; xenobiotic and cholesterol transport* (ABCA1, ABCG2)*; acetate and fatty acid activation and intracellular transport* (ACSS1, ACSS2, ACSL1, ACBP, *and* FABP3)*; fatty acid synthesis and desaturation* (ACACA, FADS1, FADS2, FASN, *and* SCD5)*; triacylglycerol synthesis* (AGPAT6, DGAT1, DGAT2, GPAM, *and* LPIN1)*; sphingolipid synthesis* (ASAHL, LASS2, OSBP, OSBPL10, OSBPL2, SGPL1, SPHK2, SPTLC1, SPTLC2, *and* UGCG)*; lipid droplet formation* (ADFP, BTN1A1, PLIN, *and* XDH)*; ketone body utilization* (BDH1, OXCT1);* and transcriptional regulation* (INSIG1, INSIG2, PPARG, PPARGC1A, PPARGC1B, SCAP, SREBF1, SREBF2, *and* THRSP)*. Of the 45 genes included in the study, 23 genes did not have complete ESTProfiles and hence could not be included for further analysis. Notably, the absence of ESTProfiles of these 23 genes does not depress the robustness of the methodology that has been employed in the present study. Clearly, as more and more ESTProfiles get submitted to UniGene, it would become possible to use the same approach for analyzing the expression patterns of different genes including those of these 23 genes. Further, though ESTProfile TPM values lack exactitude as a measure of gene expression, the differences in TPM values tend to correlate with overall expression patterns.

### 3.2. TPM_ma_/TPM_s_ and Percent Transcript Abundance: Functional Inferences

**Figure 1:**
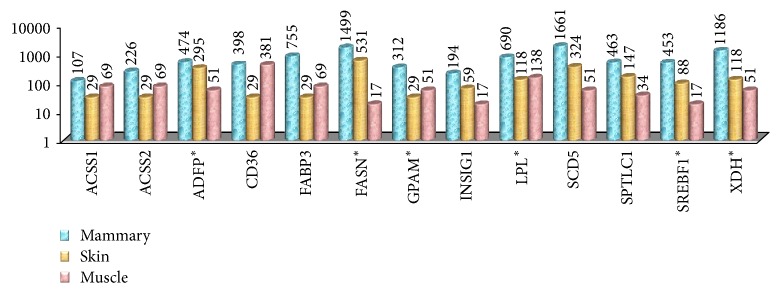
Transcript abundance of overexpressed genes. Based on TPM_ma_: TPM_s_ and *E*_*r*_ values, 13 genes appeared to be overexpressed in mammary tissue. Transcript abundance values for these genes in mammary tissue, skin, and muscle have been shown for comparison. Genes marked with “*∗*” showed preferential expression in adult-derived tissues.

Based on *E*_*r*_ and TPM_ma_: TPM_s_ values, 13 genes (*ACSS1*,* ACSS2*,* ADFP*,* CD36*,* FABP3*,* FASN*,* GPAM*,* INSIG1*,* LPL*,* SCD5*,* SPTLC1*,* SREBF1, *and* XDH*; Table 2; Figure 1) were found to exhibit higher mammary expression over skin or muscle; 6 of these 13 genes (*ADFP*,* FASN*,* GPAM*,* LPL*,* SREBF1,* and* XDH*) further showed preferential expression during adulthood.

The skin has been included for comparison because the mammary tissue is known to be modified cutaneous tissue [9] and differences in the expression pattern of genes between mammary and cutaneous tissue are likely to signify functional differences; muscle tissue has been included as a control.* ACSS1*,* ACSS2*,* ADFP*,* CD36*,* FABP3*,* FASN*,* GPAM*,* INSIG1*,* LPL*,* SCD5*,* SPTLC1*,* SREBF1, *and* XDH *had higher mammary expression over skin or muscle;* ADFP*,* FASN*,* GPAM*,* LPL*,* SREBF1,* and* XDH* showed preferential expression during adulthood and, hence, was considered most likely to be differentially expressed during milk fat synthesis.

Among genes responsible for fatty acid import into cells, both* LPL* and* CD36* appeared to have greater expression in mammary tissue.* LPL* primarily functions in the hydrolysis of triglycerides of circulating chylomicrons and very low-density lipoproteins (VLDL).* CD36* binds long-chain fatty acids and functions in their transport and also as a regulator of fatty acid transport.* LPL* showed more than 5-fold increase in TPM values in mammary tissue over cutaneous tissue whereas* CD36* showed a more than 13-fold increase. Further, the expression of* LPL* was greater in adult-derived tissues than in tissues derived from young ones. Our findings support the predication that* LPL *has higher mammary activity by virtue of high transcript abundance [10].* LPL *was the fifth most abundant transcript. Also, more than 8-fold increase in transcript abundance of* CD36* has been previously reported during* in vivo *studies [3].

Among the five genes for acetate/fatty acid activation and intracellular transport, three showed relatively higher expression in mammary tissue.* ACSS1 *showed a >3-fold increase, and* ACSS2 *showed more than 7-fold increase in transcript abundance. These findings are comparable to previous findings; Bionaz and Loor have reported a higher (~13-fold) increase in* ACSS2* over* ACSS1* (~4-fold) [3].* ACSS1* and* ACSS2* are responsible for activation of short-chain fatty acids; while* ACSS1*, primarily mitochondrial enzyme, activates acetate for energy production,* ACSS2*, the cytosolic enzyme, activates acetate for fatty acid synthesis [11]. With acetate being the chief substrate for energy production and fatty acid synthesis in the mammary tissue [9], overexpression of* ACSS1* and* ACSS2* during lactation is teleologically expected. In the same study [3],* FABP3* was the second most abundant transcript with a nearly 80-fold change in transcript abundance at 60 days of lactation. However, the relative change in transcript abundance at onset and 15, 30, 120, and 240 days of lactation ranged about 20–40. In our study,* FABP3 *showed a >26-fold increase and was also the fourth most abundant transcript among all ones considered in the study.* FABP3 *is involved in the intracellular trafficking long-chain fatty acids and their acyl coesters.

Fatty acid synthesis and desaturation per se are the most important step in milk fat synthesis. However, of the five genes studied, only two appear to be involved during the milk fat synthesis response in the mammary tissue.* FASN* that catalyzes the formation of long-chain fatty acids from acetyl-CoA, malonyl-CoA, and NADPH was the second most abundant transcript and showed ~3-fold increase in expression.* SCD5*, responsible for introducing a double bond in fatty acyl-coenzyme A at the delta 9 position, was the most abundant transcript (~15.6%) with more than 5-fold increase in mRNA expression. Bionaz and Loor have also reported* SCD5* to be the most abundant (~23%) among transcripts of genes involved in milk fat synthesis. However, in their study, the relative increase in expression has been reported to be much higher (~10–40-fold increase) [3].

*GPAM*, with more than 2% of all transcripts studied, was the only one of five genes involved in triacylglycerol synthesis found to be overexpressed (>10-fold increase). Bionaz and Loor have reported identical values of transcript abundance and relative expression of this gene [3]. Among the genes involved in sphingolipid synthesis,* SPTLC1 *appeared to be overexpressed (>3-fold) whereas the expression of* SGPL1* appeared to be downregulated at about 1/20th of cutaneous expression.

Among the genes involved in lipid droplet formation,* ADFP *and* XDH *were overexpressed with 1.6- and a 10-fold increase in relative expression, respectively, over the cutaneous tissue. Both of these genes also showed preferential expression in adult-derived tissues.* XDH* includes xanthine dehydrogenase and xanthine oxidase; the enzyme can be converted from the dehydrogenase form (D) into the oxidase form (O) irreversibly by proteolysis or reversibly through the oxidation of sulfhydryl groups.* XDH* was the third most abundant of all transcripts (>11%). Bionaz and Loor have similarly reported >7% abundance of* XDH *transcripts and about 8-fold increase in its relative expression in the lactating mammary tissue [3].

Among transcriptional regulators that drive or sustain milk fat synthesis,* INSIG1 *and* SREBF1* appeared to be overexpressed. Percent transcript abundance and relative increase in expression for the genes were about 1.8%, ~3-fold, and 4.2%, ~5-fold, respectively; 2.4- and 2.5-fold increases in the expression of these two genes have been reported previously [12]. Increase in* INSIG1* [3] and* SREBF1* [13] activities during lactation to much greater extents than being reported in the present study have also been reported earlier. A greater function of* SREBF2 *than* SREBF1* in milk fat synthesis has been hypothesized [3]. Our study could not include* SREBF2* due to insufficient information on this gene in the UniGene ESTProfile. However, based on our results,* SREBF1* is expected to play a role at least equivalent to, if not greater than,* SREBF2* in regulating the transcriptional response during milk fat production in the mammary tissue. None of the genes involved in xenobiotic and cholesterol transport and ketone body utilization appeared to be differentially expressed as part of the lactational milk fat synthesis response.

### 3.3. Gene Interaction Networks

**Figure 2:**
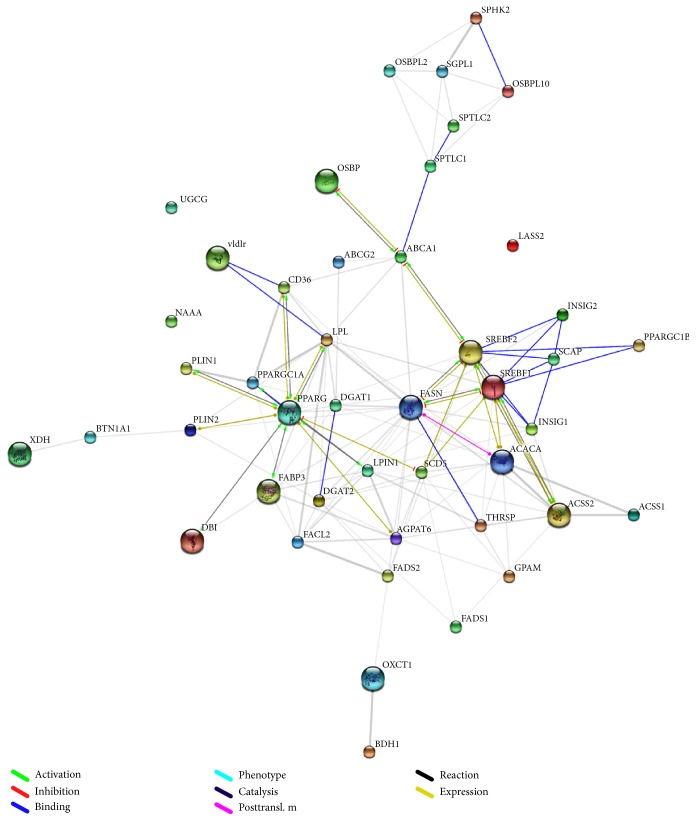
Interaction network of the genes involved in milk fat biosynthesis and secretion. STRING* v*9.1 was used to derive the network among genes involved in milk fat synthesis and secretion.* FASN* appears to be the central component in milk fat synthesis. The entire network appears to operate under two different control systems: one under* PPARG* and another under the joint control of* SREBF1 *and* SREBF2*. Three genes (*ASAHL*/*NAAA*,* LASS2,* and* UGCG*) involved in sphingolipid synthesis did not interact with any other gene/gene product in the network.

**Figure 3:**
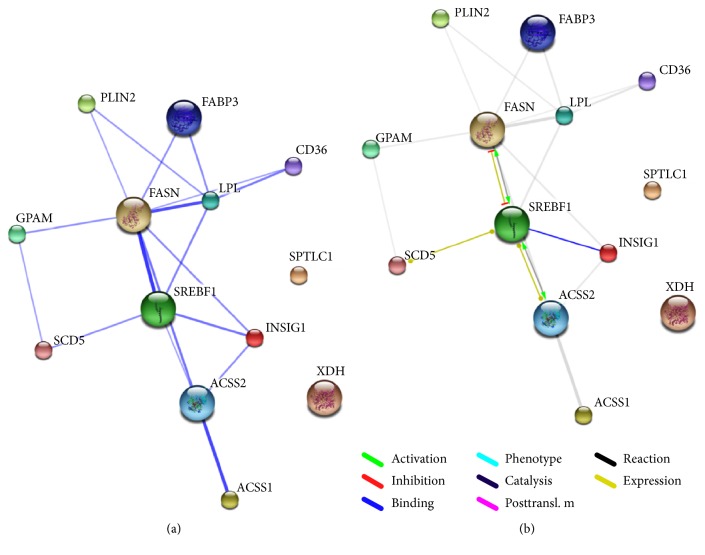
Interaction network of overexpressed genes. STRING* v*9.1 was also used to map the interaction networks between the genes that appeared to be overexpressed based on transcript abundance studies. In (a), the weight of the edges represents the confidence of the interaction; the nature of these interactions has been shown in (b).* SPTLC1 *and* XDH* did not interact with any other gene of the 11 genes.

Interaction network for all the 45 genes, obtained using STRING, has been shown in Figure 2. The interactions were further purged to map only those 13 genes that showed preferential expression in mammary tissue in UniGene ESTProfile (Figure 3); in Figure 3(a), the weight of the edges shows the strength of the interactions. The nature of these interactions has been depicted in Figure 3(b).

Network analysis shows that* FASN*,* SREBF1*,* SREBF2*,* PPARG,* and* ACSS2* are the major components of the milk fat synthesis pathway. Two subnetworks are evident: one under the predominant control of* PPARG* and the other one majorly under the joint control of* SREBF1* and* SREBF2*; both these subnetworks appear to converge at* FASN*.* SCD5*, the most abundant transcript, was the only gene under the direct control of* PPARG*,* SREBF1, *and* SREBF2*. Also, three of the four genes showing the maximum relative change in expression, namely,* FABP3*,* CD36*, and* XDH,* were chiefly under the control of* PPARG*. Thus,* PPARG*, though not found to be overexpressed based on TPM values, appears to play a major role in the transcriptional regulation of milk fat synthesis. Bionaz and Loor [3] have also advocated a role of* PPARG* in regulating the entire bovine milk fat synthesis machinery notwithstanding its downregulation and low mRNA abundance in mammary tissue. The genes involved in sphingolipid synthesis and ketone body utilization appeared to form two nearly independent clusters with sparse interaction with the rest of the network;* ASAHL (NAAA)*,* LASS2,* and* UGCG* did not interact with any other gene at all.* THRSP* did not form part of the cluster of genes involved in transcriptional regulation. While all other gene products were involved directly or indirectly in interactions with each other,* SPTLC1 *and* XDH* did not interact with any of these gene products.

**Figure 4:**
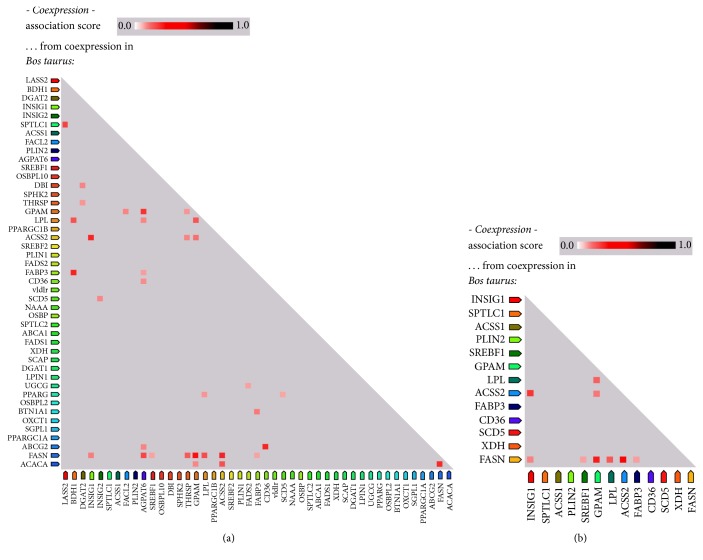
Coexpression of genes involved in milk fat biosynthesis and secretion. Coexpression pattern of the genes involved in milk fat synthesis and secretion in bovines (a) was derived from STRING. Analysis of the coexpression pattern of the thirteen genes that appeared to be overexpressed based on transcript abundance (b) showed a weak association between these genes.* FASN* shows coexpression based functional association with the maximum number of genes in both (a) and (b).

STRING was also used to determine coexpression patterns between these genes; a functional association of the gene products can be assumed if a group of genes exhibits strong coexpression. Only a low level of association could be inferred between some of the genes based on the coexpression pattern (Figure 4). Again,* FASN* appeared to be the central component of the milk fat synthesis pathway.

To conclude, in this study we have put forward a simplistic approach for determining the relative expression of genes based on their transcript abundance values in UniGene ESTProfile. Further, we used this approach for the expression profiling of genes involved in milk fat biosynthesis and secretion in bovines. Based on our findings, an updated model of the transcriptional profile of the genes involved in milk fat production by the mammary gland has been presented. For the genes studied, the results were in good agreement with the previously reported results from wetlab studies, indicating the satisfactory performance of our computational approach. Our study included cutaneous tissue as a control assuming its ontogenetic equivalence to the quiescent, nonlactating mammary gland; the congruity of our findings with those from previous studies projects this equivalence beyond the histological landscape to a biomolecular level. Previously,* SREBF2* has been upheld as the major regulator of transcription during milk fat biosynthesis, refuting the role of* SREBF1*. Our results reinstate* SREBF1* as a major transcriptional regulator, along with* INSIG1*, during the process. Using interaction network analysis of the genes, we could also show two separate transcriptional controls under* PPARG* and* SREBF*s.* FASN*,* SREBF1*,* SREBF2*,* PPARG,* and* ACSS2* were the major components of the milk fat synthesis pathway. However, expression profiles could not be studied for nearly half of the genes due to incomplete UniGene ESTProfile. Also, the inferences would have been more conclusive if UniGene ESTProfile also included information on the stage of lactation during which the mammary glands had been sampled. Thus, further studies are warranted to verify the proposed model and to fill in the research gaps in the present study.

## Figures and Tables

**Table 1:** Genes involved in milk fat synthesis and secretion. 45 genes previously reported to be involved in nine different bioprocesses (in bold) of milk fat biosynthesis and secretion [3] were studied.

Gene name	Gene product
**(1) FA import into cells**	
*CD36*	CD36 molecule (thrombospondin receptor)
*LPL*	Lipoprotein lipase
*VLDLR*	Very-Low-Density Lipoprotein Receptor
**(2) Xenobiotic and Cholesterol transport**	
*ABCA1*	ATP-binding cassette, subfamily A (ABC1), member 1
*ABCG2*	ATP-binding cassette, subfamily G (WHITE), member 2
**(3) Acetate and FA activation and intracellular transport**	
*ACBP*	Acyl-CoA binding protein (diazepam binding inhibitor)
*ACSL1*	Acyl-CoA synthetase long-chain family member 1
*ACSS1*	Acyl-CoA synthetase short-chain family member 1
*ACSS2*	Acyl-CoA synthetase short-chain family member 2
*FABP3*	Fatty acid-binding protein, heart
**(4) Fatty acid synthesis and desaturation**	
*ACACA*	Acetyl-coenzyme A carboxylase alpha
*FADS1*	Fatty acid desaturase 1 (delta-5 desaturase)
*FADS2*	Fatty acid desaturase 2 (delta-6 desaturase)
*FASN*	Fatty acid synthase
*SCD5*	Stearoyl-CoA desaturase (delta-9-desaturase)
**(5) Triacylglycerol synthesis**	
*AGPAT6*	1-Acylglycerol-3-phosphate O-acyltransferase 6
*DGAT1*	Diacylglycerol acyltransferase 1
*DGAT2*	Diacylglycerol acyltransferase 2
*GPAM*	Glycerol-3-phosphate acyltransferase, mitochondrial
*LPIN1*	Lipin 1
**(6) Sphingolipid synthesis**	
*ASAHL*	N-Acylsphingosine amidohydrolase-like
*LASS2*	LAG1 homolog, ceramide synthase 2
*OSBP*	Oxysterol-binding protein 1
*OSBPL10*	Oxysterol-binding protein-like 10
*OSBPL2*	Oxysterol-binding protein-like 2
*SGPL1*	Sphingosine-1-phosphate lyase
*SPHK2*	Sphingosine kinase 2
*SPTLC1*	Serine palmitoyltransferase, long-chain base subunit 1
*SPTLC2*	Serine palmitoyltransferase, long-chain base subunit 2
*UGCG*	Ceramide glucosyltransferase
**(7) Lipid droplet formation**	
*ADFP*	Adipose differentiation related protein (adipophilin, PLIN2)
*BTN1A1*	Butyrophilin, subfamily 1, member A1
*PLIN*	Perilipin
*XDH*	Xanthine dehydrogenase
**(8) Ketone body Utilization**	
*BDH1*	3-Hydroxybutyrate dehydrogenase, type 1
*OXCT1*	3-Oxoacid CoA transferase 1
**(9) Regulation of transcription**	
*INSIG1*	Insulin-induced gene 1
*INSIG2*	Insulin-induced gene 2
*PPARG*	Peroxisome proliferator-activated receptor gamma
*PPARGC1A*	PPAR gamma, coactivator 1 alpha
*PPARGC1B*	PPAR gamma, coactivator 1 beta
*SCAP*	SREBP cleavage activating protein
*SREBF1*	Sterol regulatory element-binding transcription factor 1
*SREBF2*	Sterol regulatory element-binding transcription factor 2
*THRSP*	Thyroid hormone responsive SPOT14

**Table 2:** Summary of results of transcript abundance studies. Of the 45 genes involved, 23 genes (S. numbers “23–45”) did not have complete UniGene ESTProfile and were precluded from further studies. Of the 22 genes studied (S. numbers “1–22”), 13 genes (in bold) appeared to be overexpressed in mammary tissue. Of these, six genes (marked with an asterisk) further showed preferential expression in adult-derived tissues. TPM: transcripts per million; ma: mammary; s: skin; mu: muscle.

S. number	Gene	TPM_ma_	TPM_s_	TPM_mu_	% transcript abundance	TPM_ma_/TPM_s_	*E*_*r*_
(1)	*ACBP*	64	118	86	0.601	0.542	0.635
(2)	*ACSL1*	172	295	363	1.616	0.583	0.526
(3)	**ACSS1**	107	29	69	1.005	3.690	2.392
(4)	**ACSS2**	226	29	69	2.124	7.793	5.052
(5)	**A** **D** **F** **P** ^*∗*^	474	295	51	4.454	1.607	3.864
(6)	**CD36**	398	29	381	3.740	13.724	3.786
(7)	*DGAT1*	32	88	17	0.301	0.364	0.827
(8)	**FABP3**	755	29	69	7.095	26.034	16.878
(9)	**F** **A** **S** **N** ^*∗*^	1499	531	17	14.086	2.823	15.777
(10)	**G** **P** **A** **M** ^*∗*^	312	29	51	2.932	10.759	8.113
(11)	**INSIG1**	194	59	17	1.823	3.288	6.126
(12)	*LASS2*	194	324	17	1.823	0.599	2.614
(13)	**L** **P** **L** ^*∗*^	690	118	138	6.484	5.847	5.407
(14)	*OSBP*	21	59	17	0.197	0.356	0.663
(15)	*PLIN*	32	29	34	0.301	1.103	1.019
(16)	*PPARG*	75	177	17	0.705	0.424	1.367
(17)	*SCAP*	21	51	17	0.197	0.412	0.713
(18)	**SCD5**	1661	324	51	15.608	5.127	12.921
(19)	*SGPL1*	10	177	34	0.094	0.056	0.129
(20)	**SPTLC1**	463	147	34	4.351	3.150	6.549
(21)	**S** **R** **E** **B** **F**1^*∗*^	453	88	17	4.257	5.148	11.712
(22)	**X** **D** **H** ^*∗*^	1186	118	51	11.145	10.051	15.288
(23)	*ABCA1*	0	0	0	0	—	—
(24)	*ABCG2*	0	0	0	0	—	—
(25)	*ACACA*	10	0	17	0.094	—	—
(26)	*AGPAT6*	593	29	0	5.572	20.448	—
(27)	*ASAHL*	0	0	17	0	—	—
(28)	*BDH1*	0	118	51	0	—	0
(29)	*BTN1A1*	744	0	0	6.991	—	—
(30)	*DGAT2*	0	88	103	0	—	0
(31)	*FADS1*	75	0	51	0.705	—	—
(32)	*FADS2*	0	0	0	0	—	—
(33)	*INSIG2*	0	0	0	0	—	—
(34)	*LPIN1*	0	0	138	0	—	—
(35)	*OSBPL10*	140	0	17	1.316	—	—
(36)	*OSBPL2*	0	88	0	0	—	—
(37)	*OXCT1*	10	0	0	0.094	—	—
(38)	*PPARGC1A*	21	0	51	0.197	—	—
(39)	*PPARGC1B*	0	0	0	0	—	—
(40)	*SPHK2*	0	0	0	0	—	—
(41)	*SPTLC2*	10	0	17	0.094	—	—
(42)	*SREBF2*	0	295	51	0	—	0
(43)	*THRSP*	0	29	69	0	—	0
(44)	*UGCG*	0	29	0	0	—	—
(45)	*VLDLR*	0	0	34	0	—	—
